# Tracing governance transitions: A qualitative framework for analysing shifts from rights-based to entrepreneurial governance regimes

**DOI:** 10.1016/j.mex.2026.104090

**Published:** 2026-08-05

**Authors:** Muhammad Alif K. Sahide, Marsleni Caezaria Daben, Nurhady Sirimorok, Truly Santika

**Affiliations:** a Forest and Society Research Group (FSRG) of Faculty of Forestry, Universitas Hasanuddin, Jl. Perintis Kemerdekaan KM.10, Tamalanrea Indah, Tamalanrea, Makassar, Indonesia; b Doctoral Program in Faculty of Forestry, Universitas Hasanuddin, Jl. Perintis Kemerdekaan KM.10, Tamalanrea Indah, Tamalanrea, Makassar, Indonesia; c Natural Resources Institute (NRI), University of Greenwich, Medway Campus, Central Avenue, Chatham Maritime Chatham, Kent, ME4 4TB, United Kingdom

**Keywords:** Governance transitions, Environmental governance, Rights-based governance, Entrepreneurial governance, Policy regime analysis, Qualitative governance methods

## Abstract

Environmental and natural resource governance rarely changes through a single policy break. Shifts are more often expressed through changing objectives, institutional incentives, evaluation indicators, evidence practices, and the roles assigned to communities. This article introduces Governance Transition Tracing (GTT) for examining these changes over time. Developed through iterative application in social forestry research, GTT integrates institutional actor mapping, policy objectives, evidence systems, and community positioning. It is intended for use in environmental governance, conservation, community forestry, and rural development, particularly where rights-based, participatory, and entrepreneurial governance logics overlap. The framework helps researchers distinguish rights-based, hybrid, and entrepreneurial governance configurations and compare governance trajectories across phases and cases.

• Traces governance through five analytical dimensions and six analytical steps.
• Combines interviews, documents, reports, and observations through cross-source triangulation.
• Supports the identification of rights-based, hybrid, and entrepreneurial governance regimes.

## Graphical abstract

**Figure:**
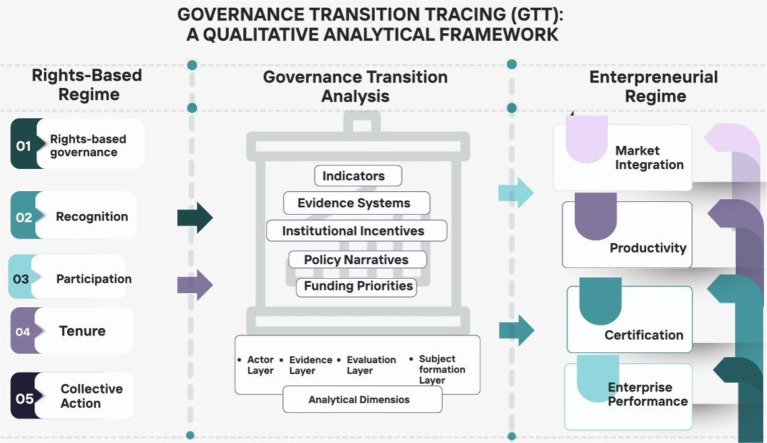

Specifications table

**Table:** 

**Subject area**	Environmental Science
**More specific subject area**	Human Ecology
**Name of your method**	Governance Transition Tracing (GTT)
**Name and reference of original method**	This method is newly developed in this study and does not adapt an existing standardised methodological protocol.
**Resource availability**	Not Applicable

## Background

Over the past three decades, reforms in natural resource governance across the Global South have sought to expand participation, strengthen social and environmental protections, and reduce highly centralised state control over forests, land, and coastal resources [[1], [2], [3]]. Many of these reforms emerged alongside decentralisation and democratisation, creating new institutional space for communities to participate in resource management. Rights-based approaches became an important part of this shift by recognising local communities as legitimate governance actors.

Rights-based governance may take the form of community forestry permits, devolved management authority, or formal recognition of customary and indigenous tenure. Beyond changing legal status, such reforms can strengthen participation, tenure security, collective action, and environmental justice [[4], [5], [6], [7], [8]]. They may also alter the political relationships between state institutions and local resource users by giving communities a more explicit role in decisions over land and resources.

Indonesia’s social forestry programme offers a useful setting in which to examine these dynamics. Through several legal schemes, the programme has expanded community access to state forest areas while pursuing livelihood improvement and participatory forest management [9,10]. However, recent studies also show that implementation remains shaped by persistent power asymmetries, informal institutions, and market influences that frequently reshape programme outcomes in practice [11,12]. Implementation, therefore, brings together objectives that do not always align [13]. Tenure recognition and community participation operate alongside state development agendas, livelihood targets, and market integration [10,11]. Communities may accept, reinterpret, or resist these arrangements, producing governance practices that cannot be described as wholly rights-based or completely market-oriented [11,13,14].

Economic performance has also become more prominent in natural resource governance settings. Government agencies and development organisations increasingly assess implementation through productivity, enterprise development, certification, market access, and other measurable outcomes [13,[15], [16], [17], [18]]. Indonesian social forestry reflects this tendency: tenure and participation remain important, but they now coexist with business development assistance, market-oriented interventions, and enterprise-based evaluation [11]. The result is seldom a complete replacement of one policy model by another. More commonly, rights-based and entrepreneurial priorities are combined within the same programme, producing hybrid governance arrangements.

Despite growing empirical evidence of such transformations, methodological guidance for systematically tracing governance transitions remains limited. Existing studies frequently document policy outcomes, institutional reforms, or implementation challenges, and rarely provide analytical procedures for identifying how governance regimes evolve through changes in policy objectives, evaluation indicators, evidence systems, and the positioning of communities within governance arrangements.

Governance Transition Tracing (GTT) was developed to address this methodological problem. It brings together institutional actor mapping, policy objective analysis, indicator tracking, evidence-system examination, and community positioning analysis within a single qualitative workflow. GTT can be applied in natural resource governance, conservation, and rural development research, while retaining an explicit concern with power and institutional change [14].

## Method details

### Workflow of governance transition tracing

GTT is a qualitative method for examining how governance arrangements change over time. It is particularly useful when rights-based and participatory commitments remain formally in place but are increasingly accompanied by enterprise development, market integration, or performance-based evaluation. GTT traces how objectives, institutional roles, indicators, evidence practices, and community positioning evolve across successive governance phases.

GTT examines governance change through multiple analytical layers by integrating institutional analysis, policy evaluation metrics, and community-level experiences to trace how governance priorities evolve over time. The framework is applicable across a wide range of natural resource governance and development contexts, including community forestry, conservation, coastal resource management, and rural development programmes where participatory governance intersects with market oriented policy reforms.

**Fig. 1:**
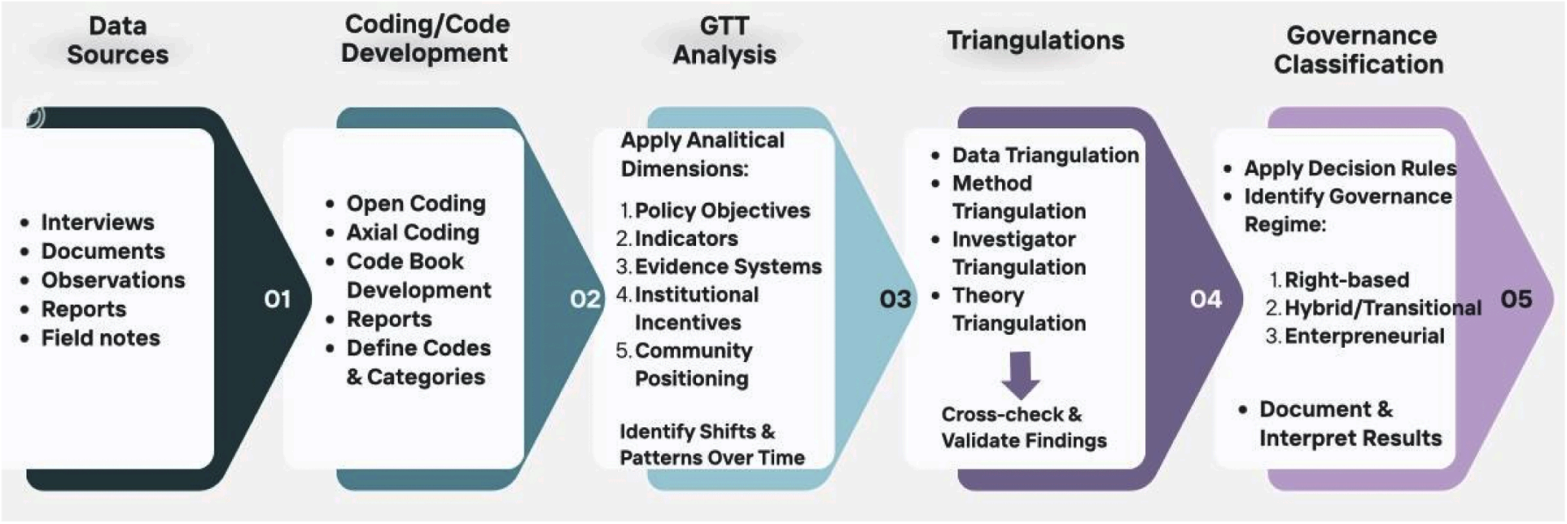
Analytical workflow of Governance Transition Tracing (GTT).

The overall analytical workflow of GTT is presented in Fig. 1, while Table 1 details each analytical step together with its guiding questions and corresponding data sources. The framework integrates multiple data sources, coding categories, analytical steps, and triangulation procedures to support the identification of governance transitions. The arrows indicate the sequential analytical process through which raw qualitative data are transformed into governance regime classifications.

**Table 1:** Workflow of Governance Transition Tracing.

Step	Analytical focus	Key questions	Data sources
1	Institutional actor mapping	Who are the key actors involved in governance and implementation?	policy documents, organisational mandates, interviews
2	Policy objective identification	What are the formal and informal goals of the programme?	programme guidelines, government reports
3	Indicator transformation tracking	How is success evaluated and how do indicators change over time?	monitoring reports, evaluation frameworks
4	Evidence system examination	What forms of knowledge are considered valid evidence?	administrative reports, policy narratives
5	Community positioning analysis	How are communities positioned within governance frameworks?	interviews, focus groups, field observation
6	Governance regime identification	What governance regime emerges from the synthesis of these dimensions?	cross-analysis of all collected data

### Data requirements and sampling strategy

GTT requires data that capture governance dynamics across institutional, policy, and community levels. The framework is designed for qualitative case studies and draws on multiple sources of evidence, including policy and regulatory documents from different implementation periods, programme monitoring and evaluation reports, interviews with institutional actors, interviews or focus group discussions with community members, and, where available, observational or archival materials.

Sampling follows a purposive and theoretically informed strategy. Researchers should select participants from different positions within the governance system, including policymakers, implementing agencies, facilitators, civil society organisations, private-sector actors where relevant, and community members. This sampling strategy supports analytical triangulation by comparing recurring patterns across actor groups and multiple data sources. The GTT consists of six analytical steps designed to guide researchers in systematically identifying governance regime transitions.

**Step 1. Institutional Actor Mapping**

The first step involves identifying the institutional actors involved in the governance system under investigation. This includes mapping both formal and informal actors involved in policy design, implementation, and evaluation. Researchers should identify actors across multiple governance levels, including:

• National policy institutions
• Regional or local government agencies
• Implementing bureaucracies
• Civil society organisations
• Private sector actors
• Community institutions or customary authorities

The purpose of this step is to understand how authority and responsibility are distributed within the governance system. Institutional actor mapping also helps identify which actors play dominant roles in producing policy narratives, evaluation criteria, and evidence used in governance decision-making. Data sources for this step may include policy documents, organisational mandates, programme guidelines, interviews with institutional actors, and participant observation in governance forums.

**Step 2. Policy Objective Identification**

The second step examines the formal and informal objectives of the governance programme. Researchers analyse how policy goals are defined, communicated, and prioritised across different stages of programme implementation. This step involves identifying whether policy objectives primarily emphasise:

• Recognition of community rights
• Institutional empowerment and participation
• Resource conservation
• Economic productivity
• Enterprise development and market integration

Changes in policy objectives often signal broader transformations in governance regimes. For example, a governance programme that initially emphasised community rights and participatory governance may later prioritise economic productivity or market competitiveness. Researchers should examine policy documents, programme guidelines, official speeches, and institutional reports to identify how policy objectives evolve over time.

**Step 3. Indicator Transformation Tracking**

Governance transitions are often reflected in changing evaluation metrics and performance indicators. This step analyses how success is measured within the governance programme. Researchers should identify:

• The indicators used to evaluate programme performance
• How these indicators change over time
• How institutions interpret and apply these metrics

Rights-based governance systems typically evaluate success through indicators such as legal recognition, community participation, and access to resources. In contrast, entrepreneurial governance regimes often introduce indicators focused on productivity, enterprise development, certification standards, or market integration. Tracking indicator transformations allows researchers to detect shifts in governance priorities even when formal policy narratives remain unchanged.

**Step 4. Evidence System Examination**

Governance programmes rely on specific forms of evidence to justify decisions, evaluate success, and guide policy implementation. This step examines how evidence is produced, interpreted, and mobilised within the governance system. Researchers should analyse:

• What types of data are considered valid evidence
• Which institutions produce and control this evidence
• Which forms of knowledge are excluded or marginalised

Evidence systems often privilege quantifiable indicators and administrative reporting while marginalising locally grounded knowledge, consistent with broader critiques of how development programmes render local realities technically governable [19]. Examining these evidence systems helps reveal how governance regimes prioritise certain forms of knowledge over others. Similar dynamics have been documented in studies of social forestry and customary forest recognition in Indonesia, where administrative indicators and formal reporting systems frequently shape policy implementation and institutional decision-making [14,20]. These dynamics illustrate how governance regimes prioritise certain forms of knowledge over others, shaping authority, legitimacy, and decision-making processes within environmental governance [21].

**Step 5. Community Positioning Analysis**

Governance transitions frequently involve changes in how communities are positioned within policy frameworks. This step analyses how communities are represented, evaluated, and governed within the programme. Researchers should examine whether communities are positioned primarily as:

• Rights holders
• Resource stewards
• Policy beneficiaries
• Entrepreneurial actors
• Market participants

Data sources for this step include interviews with community members, focus group discussions, and observations of programme implementation. Changes in community positioning often reflect deeper transformations in governance logic.

**Step 6. Governance Regime Identification**

The final step synthesises findings from the previous analytical steps in order to identify the dominant governance regime operating within the programme. Researchers compare patterns identified in:

• Institutional actor dynamics
• Policy objectives
• Evaluation indicators
• Evidence systems
• Community positioning

By triangulating these analytical dimensions, researchers can determine whether the governance system operates primarily within a rights-based framework, an entrepreneurial governance regime, or a hybrid configuration combining elements of both. The GTT framework therefore allows researchers to systematically identify governance transitions and analyse how institutional, political, and economic pressures reshape participatory resource governance programmes over time. The synthesis of these dimensions can be organised using a governance regime matrix that enables comparison across governance phases and cases (Table 2).

**Table 2:** Coding Categories for Governance Transition Analysis.

Analytical Dimension	Rights-Based Governance Codes	Entrepreneurial Governance Codes
Policy objectives	tenure security, recognition, participation, conflict resolution	productivity, enterprise development, market integration, competitiveness
Evaluation indicators	permits, institutional formation, participation rates	turnover, business certification, enterprise performance, market access
Evidence systems	legitimacy, local knowledge, community claims	economic metrics, performance reports, business outcomes
Community positioning	rights holders, resource stewards, beneficiaries	entrepreneurs, producers, market actors
Governance logic	redistribution, empowerment, inclusion	efficiency, competitiveness, enterprise growth

Researchers code interview transcripts, policy documents, and evaluation reports using these categories. Codes may be expanded according to the empirical contexts. The examples in Table 2 are intended as a guide and should be adapted to the governance and institutional setting of each study. For instance, enterprise development in Indonesia may be assessed using the KUPS (*Kelompok Usaha Perhutanan Sosial*) classification system, whereas other countries may rely on different organisational or enterprise performance indicators.

### Decision rules for identifying governance transitions

Governance Transition Tracing does not assume that governance regimes change abruptly or completely. Instead, transitions are identified when several analytical dimensions consistently point to changing governance priorities. A governance transition may be inferred when at least three of the following conditions are observed:

1. Policy objectives increasingly emphasise economic performance alongside or above rights recognition.
2. Evaluation indicators shift toward productivity, enterprise development, or market integration.
3. Evidence systems privilege economic metrics over participatory or legitimacy-based indicators.
4. Communities are increasingly expected to function as entrepreneurs, while recognition of their role as rights holders becomes less prominent.
5. Institutional incentives favour business development and performance-oriented interventions.

Where indicators associated with both rights-based and governance remain evident, the governance arrangement is classified as a hybrid regime, indicating that elements of both governance orientations continue to coexist.

### Analytical output

The results of GTT can be synthesised through a governance regime matrix, which summarises the key characteristics of different governance phases.

This matrix provides a structured way to visualise governance transitions and facilitates comparative analysis across cases.

### Practical considerations for applying the method

When applying GTT, researchers should recognise that governance transitions rarely occur through abrupt policy changes but emerge gradually through shifting evaluation criteria, funding priorities, and institutional narratives. Attention should be given to evidence systems, particularly to how indicators redefine policy priorities and programme success over time. Community-level experiences remain essential for interpreting these changes, making interviews, focus group discussions, and field observations important sources of evidence. Detailed implementation materials, including the semi-structured interview guide, analytical workflow, coding matrices, analytical decision rules, and supporting examples, are provided in Supplementary Appendices S1 and S2.

## Method validation

The GTT framework was validated through its application to Indonesia's social forestry programme in South Sulawesi. The case was selected because Indonesian social forestry represents one of the most extensive contemporary experiments in rights-based environmental governance, while previous studies have also documented the coexistence of market-oriented, participatory, and tenure-based objectives within its implementation [10,11,13,22,23]. The empirical application formed the basis of a companion research article currently submitted as co-submission in Forest Policy and Economics entitled *From Rights Recognition to Entrepreneurial Forestry: Governance Transition and Power Contestation in Indonesia’s Social Forestry*.

The validation dataset consisted of 34 semi-structured interviews with community members, social forestry facilitators, government officials, and civil society actors across three districts in South Sulawesi. The dataset was selected because it contained evidence from multiple governance levels, including community groups, facilitators, government agencies, and civil society organisations. These data were complemented by historical social forestry evaluation reports, policy documents, programme monitoring records, and field observations.

The six analytical steps were applied sequentially. Institutional actor mapping identified shifts in the relative influence of actors involved in enterprise development and programme evaluation. Policy objective analysis revealed increasing emphasis on business development and market integration. Indicator tracking demonstrated a transition from permit issuance and participation measures toward enterprise performance indicators, including Indonesia's KUPS (*Kelompok Usaha Perhutanan Sosial*) classification system, together with productivity indicators and market-oriented performance metrics. Evidence system analysis identified growing reliance on economic reporting and measurable outputs, while community positioning analysis revealed a shift from rights holders toward entrepreneurial actors.

The framework identified a consistent governance trajectory across interviews, policy documents, and evaluation reports. Comparing evidence from these different sources strengthened the analysis by confirming that similar patterns emerged across multiple forms of evidence, reducing the likelihood that the findings reflected a single dataset or institutional perspective. Coding results were also revisited across governance levels to examine whether the observed patterns remained consistent. Policy objectives, evaluation indicators, evidence systems, and community positioning all pointed to the same direction of change, providing confidence that the observed trajectory represented a broader transformation in governance.

To improve transparency and replicability, all materials were coded using the analytical categories presented in Table 2. Additional codes were introduced when empirical evidence could not be adequately captured by the initial coding scheme, and their inclusion was verified through comparison across interviews, documents, and reports. Governance classifications were assigned only when consistent patterns were evident across the analytical dimensions, ensuring that the interpretation reflected converging evidence from multiple sources rather than isolated observations.

The South Sulawesi application demonstrates that GTT can identify governance transformations that may remain obscured when analysis focuses only on policy discourse or institutional mandates. The framework also provides a replicable workflow that can be adapted to community forestry, conservation governance, fisheries co-management, land reform, and other participatory governance programmes.

## Limitations

The GTT framework supports the analysis of governance transformations in policy programmes where institutional priorities, evaluation indicators, and actor roles evolve over time. The method is particularly suitable for analysing governance systems that combine participatory frameworks with increasing pressures for economic performance, which is a common dynamic in natural resource governance and rural development programmes.

A key strength of GTT is its integration of institutional arrangements, policy objectives, evaluation indicators, evidence systems, and community experiences. By triangulating these dimensions, researchers can identify governance transitions that may not be immediately visible through policy discourse analysis alone. This helps explain how governance priorities shift across institutional and policy levels.

GTT relies on diverse qualitative sources of evidence, including policy documents, institutional reports, interviews, and focus group discussions. Where access to institutional actors or community perspectives is limited, the ability to fully apply the framework may be constrained. Researchers should also recognise that governance transitions rarely follow a simple or uniform pathway. Rights-based and entrepreneurial governance often coexist within the same programme, producing hybrid arrangements that evolve over time. In this context, GTT is intended to trace changing governance patterns and identify the coexistence of different governance logics without forcing governance into fixed categories.

The framework concentrates on governance dynamics within policy programmes and gives less attention to wider political and macroeconomic processes that may shape those dynamics. Such factors remain important and should be incorporated where they are relevant to the research context. Even with these limitations, GTT can also be applied to examine governance change across community-based resource management and rural development programmes. Its emphasis on qualitative evidence, triangulation, and comparative analysis also allows the framework to be adapted to different institutional and policy settings.

Potential areas of application include:

• Community forestry and social forestry programmes
• Conservation governance initiatives
• Coastal and fisheries co-management systems
• Land reform and tenure recognition programmes
• Rural development programmes that combine participation and market integration

In many of these contexts, policy programmes initially emphasise community participation and rights recognition but later incorporate entrepreneurial or market-oriented objectives. GTT offers a practical analytical way to examine these transformations. By focusing on observable changes in policy objectives, evaluation indicators, evidence systems, and community positioning, GTT supports the analysis of governance transitions across diverse institutional settings in the Global South.

## Ethics statements

The development and application of the Governance Transition Tracing framework involved qualitative field research that included interviews, focus group discussions, and observations with policy actors, community members, and civil society organisations engaged in natural resource governance programmes. Ethical approval and research permission were granted by the National Research and Innovation Agency (BRIN), Indonesia (768/SIP/IV/FR/10/2024, 800/KE.01/SK/09/2024, and 861/KE.01/SK/09/2025). All participants were informed about the purpose of the study prior to data collection, and informed consent was obtained before interviews and focus group discussions. Participation was voluntary, and participants were also informed that their contributions would be used solely for research purposes. No personal identifying information is reported in this article and institutional affiliations and individual identities have been anonymised to protect participant confidentiality Table 3.

**Table 3:** Example of Governance Regime Comparison.

Governance dimension	Rights-based governance	Entrepreneurial governance
Policy objective	Recognition of community rights	Economic productivity and enterprise
Evaluation indicators	Tenure recognition, participation	Productivity, certification, market readiness
Role of communities	Rights holders and collective actors	Market actors or rural entrepreneurs
Evidence system	Legal recognition, participatory legitimacy	Performance metrics and economic indicators
Governance logic	Redistribution and institutional strengthening	Market discipline and competitiveness

## CRediT author statement

MAKS: Supervision, Validation, Validity tests, Conceptualization, Writing- Reviewing and Editing. NS: Supervision, Validation, Validity tests, Conceptualization, Writing- Reviewing and Editing. MCD: Writing- Original draft preparation, Methodology. TS: Validity tests, Conceptualization, Methodology.

## Related research article

Co-submission in Forest Policy and Economics, 2026: From Rights Recognition to Entrepreneurial Forestry: Governance Transition and Power Contestation in Indonesia’s Social Forestry

## Declaration of competing interest

The authors declare that they have no known competing financial interests or personal relationships that could have appeared to influence the work reported in this paper.

## Data Availability

The data that has been used is confidential.

## Appendix Supplementary materials

Image, application 1

## References

[1] Ribot J.C. (2004).

[2] Agrawal A., Ribot J.C. (1999). Accountability in decentralization: a framework with South Asian and West African cases. J. Dev. Areas.

[3] Larson A.M., Soto F. (2008). Decentralization of natural resource governance regimes. Annu Rev. Environ. Resour..

[4] Sikor T., Lund C. (2009). Access and property: a question of power and authority. Dev. Change.

[5] Larson A.M., Barry D., Dahal G.R. (2010). New rights for forest-based communities? Understanding processes of forest tenure reform. Int. Forest. Rev..

[6] Ostrom E. (1990).

[7] Agrawal A. (2001). Common property institutions and sustainable governance of resources. World Dev..

[8] Budiman M.A.K., Christian Y., Afandy A., Prabowo B., Desmiwati D. (2025). Promoting private conservation in Patra Seroja Ecopark, Dumai Municipality, Riau Province, Indonesia. J. Penelitian Kehutanan Wallacea.

[9] Herrawan H., Sirimorok N., Nursaputra M., Mas'ud E., Faturachmat F., Sadapotto A., Supratman S., Yusran Y., Sahide M. (2022). Commoning the State forest: crafting commons through an Indonesian social forestry program. For. Soc..

[10] Moeliono M., Thuy P.T., Bong I.W., Wong G., Brockhaus M. (2017). Social forestry—why and for whom? A comparison of policies in Vietnam and Indonesia. For. Soc..

[11] Saputra I., Faturachmat F., Muin A.V.F., Sirimorok N., Sahide M.A.K. (2025). Sequencing the political forest: power, exclusion, and the corporate hijacking of social forestry in Indonesia. For. Policy. Econ..

[12] Ragandhi A., Hadna A., Setiadi S., Maryudi A. (2021). Why do greater forest tenure rights not enthuse local communities? An early observation on the new community forestry scheme in state forests in Indonesia. For. Soc..

[13] Sahide M.A.K., Fisher M.R., Erbaugh J.T., Intarini D., Dharmiasih W., Makmur M., Maryudi A. (2020). The boom of social forestry policy and the bust of social forests in Indonesia: developing and applying an access-exclusion framework to assess policy outcomes. For. Policy. Econ..

[14] Moeliono M., Sahide M.A.K., Bong I.W., Dwisatrio B., Nikolakis W., Moura da Veiga R. (2023). Social Value, Climate Change and Environmental Stewardship: Insights from Theory and Practice.

[15] Dressler W., Büscher B., Schoon M., Brockington D.A.N., Hayes T., Kull C.A., Shrestha K. (2010). From hope to crisis and back again? A critical history of the global CBNRM narrative. Environ. Conserv..

[16] Fletcher R. (2012). Using the master's tools? Neoliberal conservation and the evasion of inequality. Dev. Change.

[17] McAfee K. (2012). The contradictory logic of global ecosystem services markets. Dev. Change.

[18] Fletcher R., Büscher B. (2017). The PES conceit: revisiting the relationship between payments for environmental services and neoliberal conservation. Ecol. Econ..

[19] Li T.M. (2007).

[20] Mulyadi A., Keban Y.T., Ikhwan H., Maryudi A. (2026). Political will in decentralized forest governance: developing a typological-relational model from the formalization of customary forests in Indonesia. For. Soc..

[21] Khairina I., Rusdi M., Herman B. (2026). Agribusiness performance through innovation: the case of Arabica Bemba coffee in Enrekang. Hasanuddin Econ. Bus. Rev..

[22] Hak M.B., Nuryadin R., Fadlli M.D., Furkan A., Pahrudin P., Kurniawan D. (2026). The contribution of mangrove forest to coastal household income in East Lombok, Indonesia. Hasanuddin Econ. Bus. Rev..

[23] Sahide M.A.K., Siswandi S., Sirimorok N., Fisher M.R., Wong G.Y., Brockhaus M. (2026). The SPA-cube framework: an integrated approach for analysing power dynamics in environmental governance. MethodsX..

