# Supplementary material for: Tracing governance transitions: A qualitative framework for analysing shifts from rights-based to entrepreneurial governance regimes
Source: MethodsX. 2026 Aug 5;17:104090. doi: 10.1016/j.mex.2026.104090 (PMC13475655; doi:10.1016/j.mex.2026.104090)
Supplement: Supplementary file 1 [file mmc1.docx]

**APPENDIX S1. Semi-Structured Interview Guide for Governance Transition Tracing (GTT)**

This interview guide was developed to support the application of Governance Transition Tracing (GTT). Organised around the framework's main analytical components, it provides flexible guiding prompts that researchers can adapt to different participants and research contexts. Questions may be expanded, modified, or omitted according to the participant's role and the objectives of the study. Although the guide was developed for the empirical application presented in the companion study on Indonesia's social forestry programme, it is readily transferable to other environmental governance and rural development contexts.

Across all sections, the guiding questions are intended to capture three complementary dimensions of governance change: current governance arrangements, perceived changes over time, and the factors driving those changes. This structure enables researchers to trace transitions in policy objectives, institutional actors, evaluation indicators, evidence practices, and community positioning in a systematic yet flexible manner.

A. **Participant Background**

**Purpose:** To understand the participant's role, institutional position, and experience within the governance programme.

**Guiding questions**

- Could you briefly describe your role in the programme?
- How long have you been involved, and in what capacity?
- Which organisation, institution, or community group do you represent?
- What are your main responsibilities within the programme?
- How does your organisation or group contribute to programme implementation or decision-making?
- Which stakeholders do you interact with most frequently in your work?
- What are your organisation's or group's main priorities or expectations regarding the programme?
- Have your role or responsibilities changed over time? If so, how?

B. **Policy Objectives**

Purpose: To examine how programme objectives are interpreted, prioritised, and reconfigured over time.

**Guiding questions**

- What do you consider the main objective of the programme?
- Has this objective changed over time? If so, how?
- What activities were prioritised when the programme was first implemented?
- Which activities receive the greatest attention today?
- Which programme objectives receive the strongest institutional support or resources?
- Are there objectives that have become less visible or receive less attention than before? Why?
- Are there programme goals that remain part of official policy but are rarely implemented in practice?
- In your experience, are there important community priorities that are not adequately reflected in programme objectives?
- What factors have influenced these changes in priorities (e.g., policy reforms, funding requirements, market opportunities, political leadership, donor priorities)?
- In your view, who has been most influential in shaping these changing objectives?

C. **Institutional Actors**

Purpose: To identify the actors involved in governance, examine changes in their roles and influence, and understand how emerging governance arrangements reshape relationships among state, market, civil society, and transnational actors.

**Guiding questions**

- Which organisations or institutions currently play the most important role?
- Has the role of government changed over time? If so, how?
- What role do facilitators, extension officers, or implementing organisations play?
- How important are civil society organisations or community-based organisations?
- Are private-sector actors involved? If yes, what roles do they perform?
- Have new actors become involved over time (e.g., certification bodies, commodity buyers, financial institutions, auditors, or development partners)?
- Are there actors involved in product certification, quality assurance, value-chain development, or market access? If so, how do they influence programme implementation?
- Are international organisations, donors, or global supply-chain actors involved? How do they influence local decision-making?
- Which actors visit the field most frequently? For what purposes (e.g., monitoring, auditing, training, investment, certification, or evaluation)?
- Which actors have gained influence over time? Why?
- Which actors have become less influential? Why?
- How do these different actors interact, coordinate, or negotiate with one another?
- In your view, who has the greatest influence over important programme decisions, regardless of their formal authority?
- Are there actors who regularly visit the field from outside the local area? What roles do they perform, and how do they influence programme activities?
- Compared with the early years of the programme, have relationships among organisations become more collaborative, more market-oriented, or more dependent on external actors? Please explain.

D. **Evaluation Indicators**

Purpose: To identify how programme success is evaluated, how evaluation criteria have changed over time, and how these changes influence programme implementation.

**Guiding questions**

- How is programme success currently evaluated?
- Which indicators are considered most important?
- Have these indicators changed over time?
- Which programme outcomes receive the greatest attention during evaluation?
- What characteristics define a successful community-based initiative?
- Have programme priorities shifted towards different types of activities over time? For example, from conflict resolution, tenure recognition, or institutional strengthening towards enterprise development, product quality, certification, market access, or business performance?
- Have training, technical assistance, or capacity-building activities changed in response to these evaluation criteria? If so, how?
- Which activities receive the greatest institutional support because they contribute directly to programme performance indicators?
- Are economic indicators becoming more important than participation, rights, or environmental outcomes?
- In your view, are there important programme achievements that receive little recognition because they are difficult to measure?

E. **Evidence Systems**

**Purpose:** To examine what forms of knowledge are recognised as valid evidence for monitoring, evaluation, and decision-making.

**Guiding questions**

- What kinds of information are requested during programme monitoring?
- Are there standard reporting templates, forms, or indicators that organisations are expected to complete?
- Which reports are prepared regularly (e.g., monthly, quarterly, or annual reports)?
- Have reporting requirements changed over time? If so, what kinds of information are requested today that were not emphasised previously?
- Which types of data and indicators receive the greatest attention during programme evaluation?
- How are qualitative experiences or community perspectives documented, if at all?
- Are community experiences considered alongside administrative reports and quantitative indicators?
- Whose knowledge is most influential during programme evaluation and decision-making?
- Are there forms of knowledge or local experiences that are difficult to document within existing reporting systems?
- Are there important issues that are routinely discussed in practice but rarely appear in official reports?

F. **Community Positioning**

**Purpose:** To examine how communities are positioned within governance arrangements, how these expectations have changed over time, and how they shape community roles, responsibilities, and opportunities.

**Guiding questions**

- How are community members currently viewed within the programme? For example, are they primarily seen as:
  - Rights holders?
  - Forest managers?
  - Resource stewards?
  - Business actors?
  - Entrepreneurs?
- Have these expectations changed over time? If so, how?
- What roles or responsibilities are communities now expected to perform that were not emphasised previously?
- What kinds of knowledge, skills, or capacities are communities now expected to develop?
- Have programme activities or training shifted in ways that reflect these changing expectations? If so, how?
- Are communities encouraged to pursue particular activities (e.g., conservation, enterprise development, certification, value-chain participation, ecosystem restoration, or market engagement)? Which receive the greatest emphasis?
- In your view, what characteristics define a "successful" community or community organisation today? Has this changed over time?
- Are there community priorities or local ways of managing resources that receive less attention within the programme? Why do you think this happens?
- Do different actors (government, NGOs, private companies, donors, or community organisations) hold different expectations of local communities? If so, how?
- How have these changing expectations affected the way communities participate in governance and decision-making? How have community responsibilities changed?

G. **Governance Transitions**

**Purpose:** To identify how governance arrangements have changed over time, the processes driving these changes, and their implications for institutions, communities, and programme implementation.

**Guiding questions**

- Looking back over the programme's history, what do you consider to be the most significant changes in governance?
- When did you first notice these changes?
- What events, policy changes, or external developments do you think triggered these changes?
- Which actors or organisations were most influential in shaping these changes?
- Which actors or groups have benefited most from these changes? Why?
- Which actors or groups have faced new challenges or become less influential?
- In your view, what aspects of the programme have remained largely unchanged despite these broader shifts?
- Have programme priorities become more focused on rights, participation, conservation, enterprise development, market integration, certification, or other objectives? Please explain.
- Have these governance changes altered the way decisions are made or who participates in decision-making? If so, how?
- Are there governance practices or community priorities that have gradually disappeared or receive much less attention today?
- If you compare the programme today with its early years, what has become more important, what has become less important, and why?
- Overall, do you think the programme today represents a continuation of its original vision or a different governance approach? Why?

H. **Reflection**

**Purpose:** To encourage participants to reflect on the broader trajectory of governance change, its implications, and future directions.

Guiding questions

- Looking back, how would you describe the evolution of the programme?
- In your view, what have been the programme's most important achievements?
- What aspects of the programme have improved over time?
- Which original strengths or values of the programme should be maintained?
- What aspects remain problematic or require greater attention?
- Are there important programme objectives, governance practices, or community priorities that you believe have gradually been overlooked? Why?
- If current trends continue, how do you think the programme will evolve over the next five to ten years?
- What lessons from the programme's history should inform future policy and programme design?
- What should future policy prioritise to achieve more equitable, effective, and sustainable governance?

I. **Notes**

Not all questions are expected to be asked in every interview. Researchers should select and adapt the guiding questions according to the participant's role (e.g., policymakers, facilitators, community members, civil society organisations, or private-sector actors) while ensuring adequate coverage of the analytical components required for Governance Transition Tracing.

**APPENDIX S2.** Analytical Protocol for Governance Transition Tracing (GTT)

A. **Overview**

This appendix describes the analytical workflow used to transform qualitative evidence into an interpretation of governance transitions. GTT combines component-based analysis, temporal comparison, cross-component synthesis, and interpretive memo writing while remaining flexible across different research contexts. The workflow is intended to improve analytical transparency and reproducibility while remaining sufficiently flexible for application across different governance contexts.

B. **Analytical workflow**

The analytical workflow of GTT is organised into five sequential but iterative stages (Table S2-1). Although presented as a linear process for clarity, researchers may move back and forth between stages as new evidence emerges during analysis. The workflow is designed to facilitate systematic comparison across governance components while preserving the temporal and relational dimensions of governance change. Coding serves as an intermediate analytical step that progressively transforms empirical evidence into an integrated interpretation of governance transitions.

Table S2-1. Stage of analytical workflow of GTT

| **Stage** | **Analytical task** | **Expected output** |
| --- | --- | --- |
| Stage 1 | Organise evidence by governance component | Component-specific evidence |
| Stage 2 | Identify continuity and change within each component | Component trajectories |
| Stage 3 | Compare trajectories across components | Governance interactions |
| Stage 4 | Explain drivers and consequences | Transition dynamics |
| Stage 5 | Characterise the governance transition | Governance regime |

Each analytical stage builds progressively on the outputs of the preceding stage. The five stages function as an integrated analytical process in which each stage builds on the outputs of the previous stage. Although presented sequentially, researchers may revisit earlier stages whenever new evidence, contradictory findings, or alternative interpretations emerge during analysis.

**Stage 1. Organise evidence by governance component**

**Objective:** To organise interview transcripts, policy documents, reports, and field notes according to the six analytical components of GTT.

Researchers should:

- read all transcripts
- identify passages related to objectives
- identify passages related to actors
- identify passages related to indicators
- identify passages related to evidence practices
- identify passages related to community positioning
- allow multiple coding where appropriate

**Output:** Component-specific evidence database.

**Stage 2. Identify continuity and change**

**Objective:** To examine how each analytical component changes across time.

Researchers compare:

- earlier period
- transition period
- recent period

Questions guiding analysis:

- What remained stable?
- What changed?
- What became more important?
- What became less visible?
- Which new practices emerged?

**Output:** Trajectory for each component.

**Stage 3. Cross-component synthesis**

**Objective:** To identify relationships among analytical components that cannot be understood when each component is examined independently. At this stage, researchers compare trajectories across policy objectives, institutional actors, evaluation indicators, evidence systems, and community positioning to identify coherent patterns of governance change.

Researchers should examine questions such as:

- Do changes in policy objectives coincide with changes in evaluation indicators?
- Do new governance actors introduce different evidence requirements?
- Have changing evaluation systems altered community expectations?
- Do multiple components point towards the same governance trajectory?
- Are contradictions observed across different components?

**Output:** Cross-component relationships that explain how multiple governance processes interact to produce broader governance transitions.

**Stage 4. Explain drivers and consequences**

**Objective:** To explain why governance transitions occur and to examine their implications for governance actors, institutional arrangements, programme implementation, and community experiences. At this stage, researchers move beyond describing patterns of change and begin interpreting the mechanisms that have produced those changes.

Researchers should examine whether observed transitions are associated with:

- policy reforms
- political leadership
- organisational restructuring
- donor priorities
- market incentives
- certification systems
- technological change
- environmental crises
- changing funding arrangements
- evolving community aspirations

Guiding analytical questions include:

- What factors appear to explain the observed transition?
- Are multiple drivers operating simultaneously?
- Which actors benefit from the transition?
- Which actors experience new constraints?
- What intended and unintended consequences emerge?
- Are the observed changes temporary or institutionalised?

**Output:** An evidence-based explanation of the drivers, mechanisms, and consequences underlying governance transitions.

**Stage 5. Characterise governance transition**

**Objective:** To synthesise findings from all analytical components into an overall interpretation of governance change. Researchers synthesise findings across all analytical components to determine whether the observed trajectories indicate continuity, gradual adaptation, institutional layering, displacement, hybridisation, or broader governance transformation.

Researchers synthesise evidence regarding:

- governance trajectory
- dominant governance logic
- institutional continuity
- institutional layering
- displacement
- hybrid governance
- entrepreneurialisation
- rights-based governance
- market-oriented governance

Researchers should ask:

- What overall governance trajectory emerges?
- Do different analytical components indicate a consistent pattern?
- Are observed transitions incremental or transformative?
- Which governance principles become dominant?
- Are previous governance arrangements replaced or incorporated into new ones?

**Output:** A coherent interpretation of the governance transition supported by evidence from multiple analytical components.

Before developing an overall interpretation, researchers should assess whether evidence from the different analytical components consistently indicates governance change. The checklist (Table S2-2) is intended as a practical quality-control tool and is not designed to generate numerical scores.

**Table S2-2.** Example of Governance Transition Checklist

| **Analytical question** | **Yes** | **No** | **Evidence** |
| --- | --- | --- | --- |
| Have programme objectives changed? | □ | □ |  |
| Have institutional actors changed? | □ | □ |  |
| Have evaluation indicators changed? | □ | □ |  |
| Has evidence production changed? | □ | □ |  |
| Has community positioning changed? | □ | □ |  |
| Do these changes indicate a coherent transition? | □ | □ |  |

C. **Coding Matrix**

Table S2-3 illustrates one possible application of the GTT coding process. Researchers begin with verbatim quotations, assign descriptive initial codes, classify them within the analytical components, identify their temporal orientation, and develop broader interpretations of governance transitions. The coding process may be adapted according to the objectives, empirical setting, and available evidence of each study.

**Table S2-3.** Example Coding Matrix

| **Raw quotation** | **Initial code** | **Analytical component** | **Temporal orientation** | **Emerging pattern** | **Interpretation** |
| --- | --- | --- | --- | --- | --- |
| "Earlier we discussed participation..." | participation focus | Policy Objectives | Earlier phase | Rights-based priorities | Rights-oriented governance |
| "We used to discuss tenure, now most meetings are about marketing." | shift in priorities | Policy Objectives | From earlier rights focus to recent market orientation | Entrepreneurial priorities | Entrepreneurial governance |
| "Most reports now focus on production targets." | production indicators | Evaluation Indicators | Recent | Performance-based evaluation | Performance governance |
| "Community meetings are rarely mentioned in reports." | undocumented participation | Evidence Systems | Persistent omission | Documentary silence | Administrative dominance |
| "Now we are expected to become entrepreneurs." | entrepreneurial subject | Community Positioning | Emerging | Entrepreneurial subject formation | Subject repositioning |

D. **Cross-component Matrix**

Governance transitions rarely become visible within a single analytical component. Instead, they emerge through consistent patterns observed across multiple components. The cross-component matrix therefore enables researchers to compare findings from policy objectives, institutional actors, evaluation indicators, evidence systems, and community positioning in order to identify broader governance trajectories. Table S2-4 illustrates how evidence from different components can be integrated into a single analytical interpretation.

**Table S2-4.** Illustrative Cross-component Matrix

| **Policy objectives** | **Institutional actors** | **Evaluation indicators** | **Community positioning** | **Interpretation** |
| --- | --- | --- | --- | --- |
| enterprise development | private-sector buyers, certification bodies, development partners | production targets | entrepreneurs | entrepreneurial governance |
| rights recognition | government agencies, community organisations | tenure indicators | rights holders | rights-based governance |

***Analytical memo***

Analytical memos provide an intermediate step between coding and interpretation. They allow researchers to document emerging patterns, record analytical reflections, evaluate alternative explanations, and justify interpretive decisions throughout the analytical process. Memo writing therefore improves transparency, reflexivity, and consistency during Governance Transition Tracing.

**Illustrative Analytical Memo**

1. **Observation.** Interview participants consistently reported that discussions increasingly focused on enterprise development, certification, and market access.
2. **Supporting evidence.** This pattern appeared across interviews with government officials, facilitators, and community organisations and was reinforced by annual reports emphasising production performance and business development.
3. **Possible explanation.** Recent policy reforms and donor priorities increasingly rewarded measurable economic outcomes and market-oriented activities.
4. **Alternative explanation.** The observed emphasis may also reflect improvements in reporting practices and documentation, without necessarily indicating a substantive shift in governance priorities.
5. **Implication for governance transition.** These observations suggest a gradual transition from a rights-oriented governance regime towards entrepreneurial governance, accompanied by changes in evaluation practices and community expectations.
E. **Analytical Decision Rules**

Objective: Analytical decision rules provide explicit guidance for interpreting qualitative evidence consistently throughout Governance Transition Tracing. The decision rules provide guiding principles that promote systematic reasoning, analytical transparency, and consistent interpretation throughout GTT.

Researchers are encouraged to apply the following principles throughout the analytical process:

- **Decision rule 1.** A governance transition should only be inferred when evidence from multiple analytical components indicates a coherent pattern of change.
- **Decision rule 2.** Changes identified from interviews should be compared with documentary evidence whenever possible.
- **Decision rule 3.** Conflicting evidence should be retained and systematically evaluated to ensure a balanced interpretation of governance transitions.
- **Decision rule 4.** Individual quotations should not be interpreted in isolation but considered alongside broader institutional patterns.
- **Decision rule 5.** The final interpretation should synthesise findings across all analytical components to develop a robust and evidence-based interpretation of governance transitions.
- **Decision Rule 6.** No governance transition should be inferred solely from documentary evidence without considering participant perspectives.
- **Decision Rule 7**. Interpretations should recognise that continuity and change often coexist within evolving governance arrangements.

F. **Closing Note**

The analytical workflow presented in this appendix is intended to enhance the transparency, consistency, and reproducibility of GTT. Researchers may adapt specific procedures according to their empirical context, available data sources, and research objectives while maintaining the core analytical logic of the framework. This logic integrates component based analysis, temporal comparison, cross component synthesis, analytical memo writing, and evidence informed interpretation to support systematic analysis of governance transitions across different research settings.

Table S2-5 summarises the main sources of qualitative evidence commonly used in Governance Transition Tracing and their analytical contributions. Although individual studies may draw on different combinations of data, integrating multiple sources strengthens the interpretation of governance transitions by enabling triangulation across policy, institutional, and community perspectives.

**Table S2-5.** Common Data Sources and Their Analytical Contributions in Governance Transition Tracing

| **Data source** | **Possible contribution** |
| --- | --- |
| Interviews | Perceptions, experiences, governance change |
| Policy documents | Formal objectives and institutional arrangements |
| Monitoring reports | Evaluation indicators and evidence practices |
| Meeting minutes | Decision-making processes |
| Field observations | Everyday implementation |
| Archival documents | Historical comparison |

Although this analytical workflow was developed through research on Indonesia's social forestry programme, it can be applied to a wide range of environmental governance, conservation, rural development, and natural resource management contexts where governance arrangements evolve over time.
